# Quantitative low mechanical index contrast-enhanced endoscopic ultrasound for the differential diagnosis of chronic pseudotumoral pancreatitis and pancreatic cancer

**DOI:** 10.1186/1471-230X-13-2

**Published:** 2013-01-03

**Authors:** Dan Ionuţ Gheonea, Costin Teodor Streba, Tudorel Ciurea, Adrian Săftoiu

**Affiliations:** 1 Research Center of Gastroenterology and Hepatology, University of Medicine and Pharmacy, Craiova, 200349, Romania

## Abstract

**Background:**

Second-generation intravenous blood-pool ultrasound contrast agents are increasingly used in endoscopic ultrasound (EUS) for characterization of microvascularization, differential diagnosis of benign and malignant focal lesions, as well as improved staging and guidance of therapeutic procedures.

**Methods:**

The aim of our study was to prospectively compare the vascularisation patterns in chronic pseudotumoral pancreatitis and pancreatic cancer using quantitative low mechanical index (MI) contrast-enhanced EUS. We included 51 patients with chronic pseudotumoral pancreatitis (n = 19) and pancreatic cancer (n = 32). Perfusion imaging started with a bolus injection of Sonovue (2.4 ml), followed by analysis in the early arterial (wash-in) and late venous (wash-out) phase. Perfusion analysis was performed by post-processing of the raw data (time intensity curve [TIC] analysis). TIC analysis was performed inside the tumor and the pancreatic parenchyma, with depiction of the dynamic vascular pattern generated by specific software. Statistical analysis was performed on raw data extracted from the TIC analysis. Final diagnosis was based on a combination of EUS-FNA, surgery and follow-up of minimum 6 months in negative cases.

**Results:**

The sensitivity and specificity of low MI contrast enhanced EUS using TIC were sensitivity and specificity of low MI contrast enhanced EUS using TIC analysis were 93.75% (95% CI = 77.77 - 98.91%) and 89.47% (95% CI = 65.46 - 98.15%), respectively. Pseudotumoral chronic pancreatitis showed in the majority of cases a hypervascular appearance in the early arterial phase of contrast-enhancement, with a dynamic enhancement pattern similar with the rest of the parenchyma. Statistical analysis of the resulting series of individual intensities revealed no statistically relevant differences (p = .78). Pancreatic adenocarcinoma was usually a hypovascular lesion, showing low contrast-enhancement during the early arterial and also during the late venous phase of contrast-enhancement, also lower than the normal surrounding parenchyma. We found statistically significant differences in values during TIC analysis (p < .001).

**Conclusions:**

Low MI contrast enhanced EUS technique is expected to improve the differential diagnosis of focal pancreatic lesions. However, further multicentric randomized studies will confirm the exact role of the technique and its place in imaging assessment of focal pancreatic lesions.

## Background

Differentiating pancreatic adenocarcinoma from other pancreatic masses remains still challenging with current imaging techniques. Because histological assessment of the pancreas is difficult in most of the cases, different gold standards have been used to establish the optimum number of EUS criteria for diagnosis of benign or malignant disease [1].

Contrast Enhanced Harmonic Endoscopic Ultrasound (CEH-EUS) was recently proposed as a suitable tool the differential diagnosis of pseudotumoral chronic pancreatitis and pancreatic cancer, a new generation method with high resolution [2,3]. Furthermore, it has the added benefit of biological material sampling without risk of tumor seeding. Assessment of tumor vascularity by contrast enhanced EUS was limited until recently due to the inability to use second-harmonic or pulse inversion techniques, since EUS probes have high frequencies from 5 to 12 MHz. Second generation contrast agents and recent advances of new ultrasound systems now allow better visualization of intralesional vascular signals and indicate blood flow patterns of normal and diseased tissue. Because it has a better resolution than transabdominal ultrasound, CEH-EUS can thus represent the best way to quantify the tumor vasculature in a minimally invasive manner and with high accuracy [2]. The European Union approved second-generation contrast agent used in the most of the examinations is SonoVue (Bracco SpA, Milan, Italy). It contains phospholipid-stabilized microbubbles of sulfur hexafluoride (SF6), which are stable and resistant to pressure. Contrast microbubbles are restricted inside of blood vessels and do not pass into the extravascular compartment. They persist in the blood until they are eliminated by the lungs in the expired air [4].

The hypovascular aspect of the pancreatic lesions under CEH-EUS seems to be highly sensitive and specific for adenocarcinoma in several published studies [5,6]. Furthermore, the lesion size and margins are better visualized, as well as the relationship with peripancreatic arteries and veins. Focal lesions in chronic pseudotumoral pancreatitis are reported to have similar or hyperenhancement features as compared to the normal pancreatic parenchyma [7].

Dynamic CEH-EUS techniques are particularly suitable for measurement of focal lesions perfusion. Because just visual appreciation can sometimes introduce diagnosis bias, there were described some computed post-processing techniques (rise time and mean transit time, peak intensity, and area under the curve etc.) [4] which can accurately describe and calculate perfusion parameters in order to appreciate the hyper- or hypoenhancement pattern.

## Methods

The aim of our study was to prospectively compare the vascularization patterns in chronic pseudotumoral pancreatitis and pancreatic cancer using quantitative low mechanical index CEH-EUS.

We prospectively included all patients undergoing CEH-EUS as part of the investigatory routine for either chronic pancreatitis or pancreatic adenocarcinoma at the Research Center of Gastroenterology and Hepatology of Craiova. We excluded all patients with low quality examinations (n = 3), thus resulting a total of 51 patients with either chronic pseudotumoral pancreatitis (n = 19) or pancreatic cancer (n = 32). Final diagnosis was based on a combination of EUS-FNA, surgery and follow-up of minimum 6 months in cytology or histology negative cases. All patients signed informed consent forms and all study procedures were in conformity with the Helsinki Declaration, also receiving all necessary approvals from the Ethic Committees of the University of Medicine and Pharmacy of Craiova.

Patients were examined by two experienced examiners (either AS or DIG). All the patients received propofol sedation under the supervision of an anesthesiologist. EUS examination equipment included a Hitachi Preirus (Hitachi Medical Corp., Tokyo, Japan) ultrasound system coupled with the EG 3830 linear endoscope (Pentax, Hamburg, Germany). Perfusion imaging started with a bolus injection of Sonovue (2.4 ml), followed by analysis in the early arterial (wash-in) and late venous (wash-out) phase. All the examinations were digitally recorded. All image settings were maintained identical throughout the investigations. We used a MI of 0.2 for all patients. The examination ended with EUS-guided FNA, with 3 passes performed. All Time intensity curve (TIC) analysis was performed by trained personnel under their supervision during offline post-processing, as described below. We manually exported recordings of all contrast-enhanced EUS procedures for each patient in uncompressed audio-video interleaved (AVI) format at six frames per seconds, for offline processing. Afterwards, movies were loaded in the commercially available image processing software Image Pro Plus (Media Cybernetics, USA) version 7.0 in order to post-process the movies and perform the TIC analysis. As full-frame, uncompressed .AVI files were exported from the US machine, we did not encounter any image artifacts. The software used for post-processing equalized the brightness, contrast and gamma levels on a frame-by-frame basis, thus linearizing the recording. We then performed an automated frame-by-frame median intensity tracking on two regions of interest (ROIs) – one corresponding to the lesion, and one chosen from normal surrounding parenchyma – obtaining TIC data in graphical and numerical form. The TIC obtained for the normal ROI was later set as reference track in the imaging software, thus obtaining relative values for the tumoral TIC as a single data row. The whole lesion was taking into account when selecting the corresponding ROI. We used an irregular shape to trace the contour of the lesion whenever possible. Otherwise, we chose a significant portion of the lesion (in several chronic pancreatitis cases). We selected a circular area corresponding to healthy parenchyma, varying it in size on a case-by-case basis, with a minimum radius of 2 cm. The resulting data string was exported in commercial spreadsheet software for plotting and statistical analysis. We further indexed and performed basic statistical analysis on the two sets of data. We calculated peak intensities (Imax), time to peak (TTP) and area under the curve (AUC) for the resulting track. As we opted for a relative TIC representation, peak intensity for the pancreatic adenocarcinoma TIC was considered the absolute minimum intensity reached. The Mann–Whitney non-parametric test was used for assessing the differences between the enhancement patterns of the two pathologies, as described by the consecutive intensities variances. P values below 0.05 were considered significant.

## Results

An overview of the most important parameters of our study lot is presented in Table 1.

**Table 1:** Overview of the most important studied parameters

	**Women (%)**	**Age (interval)**	**Signal intensity compared to parenchyma on entire TICs (interval)**	**Correct TIC**-**based diagnosis (%)**
Pseudotumoral pancreatitis	12 (63)	45 (32 –73)	2.7 (0.8 – 6.9)	17 (89.5)
Pancreatic adenocarcinoma	14 (44)	53 (41 – 82)	−18.4 (−30.1 – 0.17)	30 (93.75)

Mean age and gender repartition in each studied pathology. Median signal intensities for pseudotumoral pancreatitis and pancreatic adenocarcinoma resulting TICs. Number of correct classifications based on TIC analysis only.

Overall, the sensitivity and specificity of low MI contrast enhanced EUS using TIC analysis were 93.75% (95% CI = 77.77 - 98.91%) and 89.47% (95% CI = 65.46 - 98.15%), respectively. The positive predictive value of TIC analysis was 93.75% (95% CI = 77.78 – 98.91%) and the negative predictive value was 89.47% (95% CI = 65.46 – 98.15%). Positive diagnosis based only on TIC analysis was achieved in 47 of the 51 patients, with two misdiagnosed cases from each category.

### TIC analysis parameters and features

**Figure 1:**
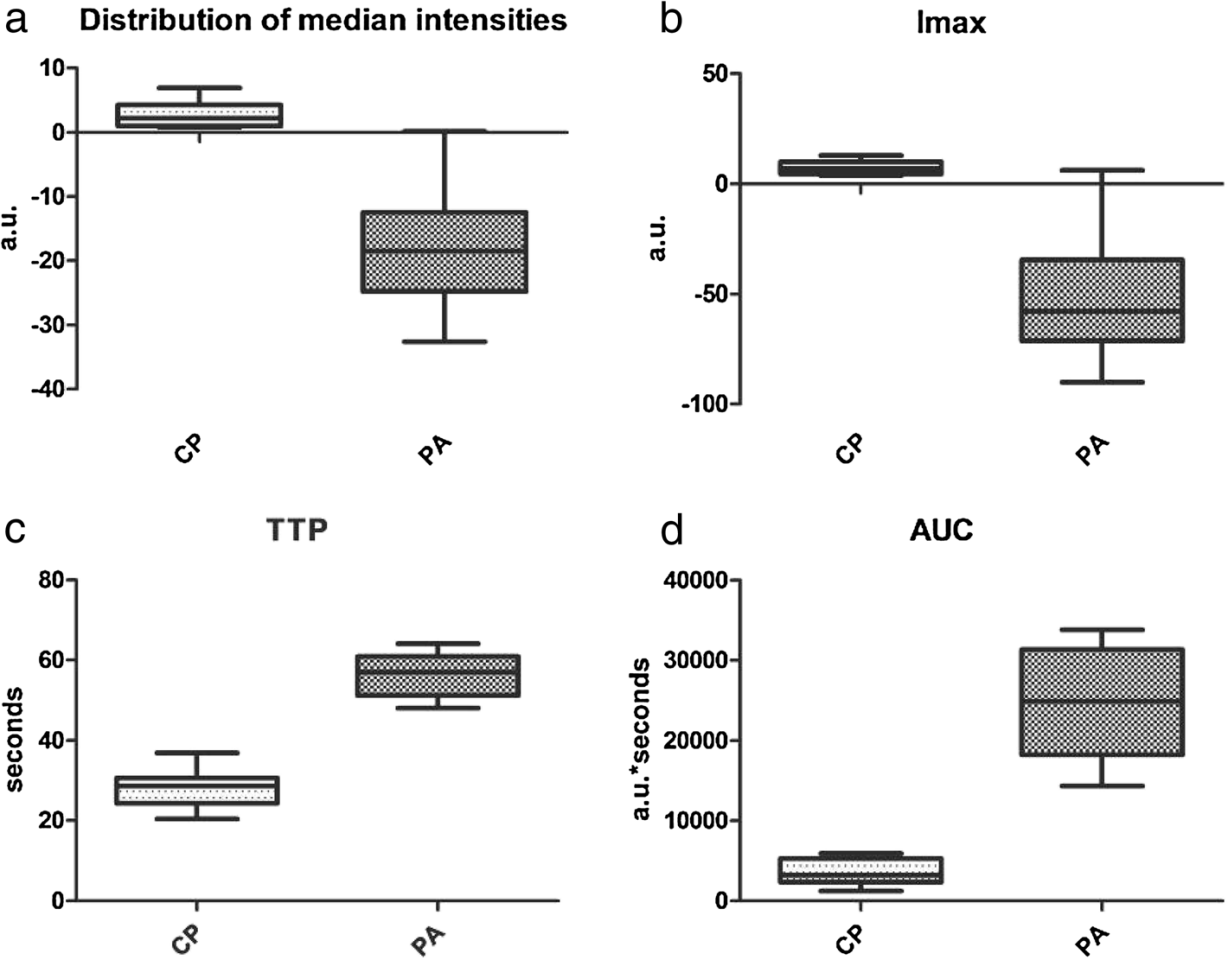
**Statistical distribution of TIC**-**related parameters within the two lots.** (**a**) Distribution of median intensities between the two pathologies. (**b**) Distribution of the maximum intensities within the two lots. (**c**) Median times to peak for the two pathologies. (**d**) Area under the curve for the two corresponding TICs. Legend: a.u. = arbitrary units; CP = chronic pancreatitis; PA = pancreatic adenocarcinoma; Imax = maximum intensity; TTP = time to peak; AUC = area under the curve.

By selecting the parenchymal ROI intensity track as reference, we obtained a single relevant TIC as the difference between the two original ones. Results are expressed as means and intervals in Table 2. Overall median intensities of the two TICs were significantly different between the two groups (p < 0.0001) (Figure 1a). We thus obtained a median Imax value of 11.4 for pancreatic adenocarcinoma, compared to −54.1 for pancreatic adenocarcinoma ROIs (Mann-Withney p < 0.0001) (Figure 1b). Time needed for the contrast agent varied for both pathologies and was significantly lower in cases of pseudotumoral pancreatitis compared to pancreatic adenocarcinoma (27.87 seconds *versus* 56.19 seconds, p < 0.0001) (Figure 1c). AUC corresponding to pseudotumoral pancreatitis TIC was also significantly lower from the AUC of pancreatic adenocarcinoma TICs, as the intensities reached were similar to the baseline parenchymal values (p < 0.0001) (Figure 1d).

**Table 2:** Mean maximum signal strengths, times to peak and areas under the curve calculated for the resulting TIC

	**Imax (interval)**	**TTP (interval)**	**AUC (interval)**
Pseudotumoral pancreatitis	11.4 (3.8 – 12.9)	27.87 (20.4 – 36.9)	3627 (1247 – 5891)
Pancreatic adenocarcinoma	−54.01 (−90.1 – 6.2)	56.19 (48.6 – 64.1)	24490 (14322 – 33789)

Imax = maximum intensity, expressed as arbitrary units; TTP = time to peak, expressed in seconds; AUC = area under the curve, expressed as a.u*seconds.

### Chronic pseudotumoral pancreatitis

**Figure 2:**
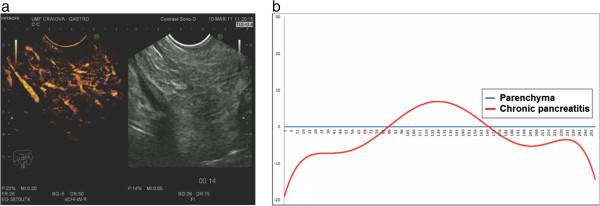
**Example of CEH**-**EUS and TIC of pseudotumoral chronic pancreatitis.** (**a**) Pseudotumoral chronic pancreatitis. (**b**) Graphical representation of the TIC trend line for the ROI corresponding to the inflammatory mass referenced to the parenchyma baseline.

Unlike malignant tumors of the pancreas, focal mass-forming pancreatitis had similar enhancement to that of the normal surrounding parenchyma (Figure 2a and 2b). Statistical analysis of the resulting series of individual intensities revealed no statistically relevant differences (p = 0.78) between the two ROIs, thus proving the isoenhancing tendency. In 17 cases of pseudotumoral chronic pancreatitis we could observe a hypervascular appearance in the early arterial phase of contrast-enhancement, with a dynamic enhancement pattern similar with the rest of the parenchyma. The two cases with unusual TIC representation showed marked hypoenhancement, similarly to PA.

### Pancreatic adenocarcinomas

**Figure 3:**
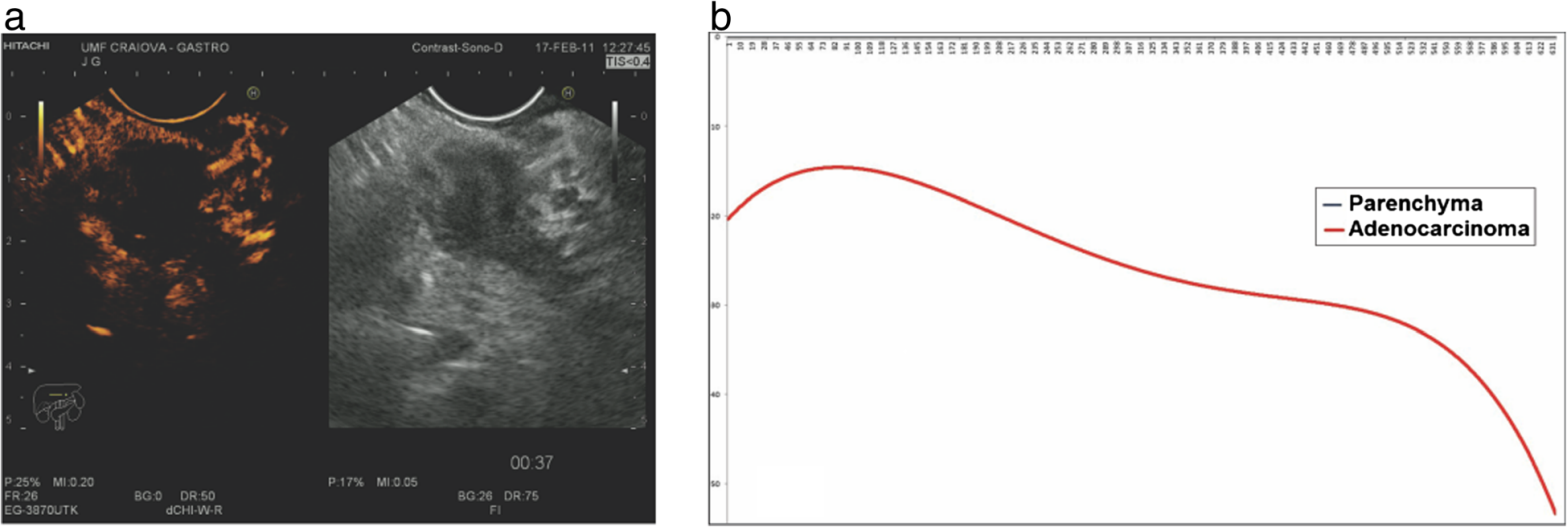
**Example of CEH**-**EUS and TIC of pancreatic adenocarcinoma.** (**a**) Pancreatic adenocarcinoma. (**b**) Graphical representation of the TIC trend line for the ROI corresponding to the tumor referenced to the parenchyma baseline.

A total of 30 pancreatic adenocarcinomas showed low contrast enhancement in both arterial and in late phases, characteristic to hypovascular lesions (Figure 3a and 3b). The remaining two cases showed initial hyper enhancement followed by rapid depletion of contrast agent then stabilization near to parenchyma values. In all other investigations, Mann–Whitney comparison of the two series of values corresponding to the tumoral and parenchyma ROIs showed statistically relevant differences between the two TICs (p < 0.001).

## Discussion

Differentiation of pseudotumoral pancratitis and pancreatic adenocarcinoma by imagistic methods has always been difficult [8,9]. Inflammatory masses present in chronic pancreatitis, called ‘pseudotumoral pancreatitis” show similar aspects during CT and MRI evaluations, thus other imaging techniques were proposed for investigating the pancreatic pathology [10]. CEH-EUS can provide essential information regarding pancreatic masses, as it can distinguish between different vascular patterns specific to each tumor. This greatly aids the differential diagnostic efforts of the physician before submitting the patient to more invasive investigations such as fine needle EUS aspiration. This in turn greatly improves the chances of targeted surgical interventions where needed, earlier and at lower costs. It is currently acknowledged that pancreaticoduodenectomy or other major pancreatic surgeries are still associated with important morbidity, with mortality rates ranging from 5 to 10% in specialized centers [11-13], with even higher numbers in smaller referral centers [11].

Several advantages of CEUS over CT and MRI examinations can be identified. First of all, it can be performed immediately, without any preliminary laboratory testing. It operates in real time so that rapid changes in enhancement can be observed and quantified. The US contrast media are not nephrotoxic, do not interact with the thyroid gland and anaphylactoid reactions are extremely rare [14]. The incidence of severe hypersensitivity or anaphylactoid reactions is lower than with current X-ray agents [15].

The mechanical index is an important parameter that influences microbubble behavior in contrast ultrasound imaging.

High mechanical index procedure in conjunction with contrast agents was initially used as color or power Doppler signal enhancement technique. One of the main disadvantages is the presence of artifacts, including flash and blooming artifacts. Furthermore they strongly disrupt and dissolve the microbubbles [4]. The new ultrasound systems use contrast-specific software modes and low mechanical index examination (between 0.08 and 0.3). CEH-EUS is a relatively new method with accepted superiority over the high mechanical index US and EUS [16].

The contrast enhanced ultrasound technique has some limitations: 10% of the pancreatic carcinomas are hypervascularised and in some cases of chronic pseudotumoral pancreatitis areas of necrosis induces clear artifacts due to lack of enhancing. These important limitations suggest that a combination of non-invasive methods (CEH-EUS, EUS elastography) can increase the accuracy of the diagnosis.

Second generation ultrasound micro-bubble contrast agents such as Sonovue showed superior safety profiles to CT or MRI contrast agents [14]. Their wide availability, relatively low costs and virtually non-existing contraindications makes them extremely useful in both external applications such as Contrast-Enhanced trans-abdominal Ultrasound (CEUS) and contrast-enhanced EUS. However, visual assessment remains subjective to the experience of the investigator, as his impression on brightness and contrast variations are relative and cannot be compared to other subsequent investigations. Thus, the need for accurate quantification of perfusion patterns in tumor masses rose from the necessity of better characterizing lesions and unifying investigatory results.

In our study, we investigated, for the first time as far as we know, the usefulness of time intensity curve (TIC) analysis in CEH-EUS application for the imaging of pancreatic adenocarcinoma and pseudotumoral pancreatitis. The presented method proved to be efficient in differentiating the two pathologies, correctly diagnosing a total of 47 cases out of the total of 51. Two cases were misdiagnosed from each pathology, showing superior accuracy in recognizing malignant masses. We obtained a sensitivity of 93.75% and specificity of 89.47% for TIC-based analysis alone. Various studies showed high sensitivity values for CEUS compared to CT when differentiating between pancreatic adenocarcinoma and pseudotumoral pancreatitis, with sensitivities between 94% and 98% and accuracies as high as 100% [17-19].

Our method implied the use of a versatile, commercially available software for quantifying perfusion data, followed by statistical analysis. Since the software was not specifically designed for TIC analysis, it may lack immediate clinical application; however, in our study, using its built-in automation ability, we could simplify the processing, once the operator selected the two ROIs. The statistical analysis was also automated through the use of external scripting, greatly reducing the time needed for the comparison, to 5 ± 2 minutes.

The quantification software provided measurements in arbitrary units, as median frame intensity for each frame of the AVI movie loaded. We opted for an offline quantification solution as it allows the processing of virtually any investigation, as long as it is provided in a popular digital movie format. This approach also permitted us to semi-automate the process of plotting the TICs and exporting raw numerical data to external spreadsheets, by taking advantage of the extensive macro abilities built in the software.

One element of novelty in our study was the attempt to statistically analyse the differences between the two raw data streams obtained before referencing the tracks between them. Paired analysis between the tumoral ROI and the corresponding parenchymal ROI thus revealed statistically relevant differences in pancreatic adenocarcinoma cases, due to the low vascularity and consecutively divergent curves obtained, compared to similar uptake patterns in pseudotumoral pancreatitis cases, resulting in minimal differences between the two rows of values (p = 0.78).

When selecting intensity-related parameters, we opted for expressing TICs as relative to one another. Hence, we considered the parenchymal ROI as “reference track” and expressed the mass-related TIC by subtracting the values, thus obtaining a single TIC which better reflected the variation in maximum intensities and times to reach peaks and were better suited for the comparison of areas under the curves. We obtained low negative values for pancreatic adenocarcinomas (with a maximum low value of −90.1 and a mean of 54.01), compared to values closer to the reference track for pseudotumoral pancreatitis. This is mainly due to the low vascularization of adenocarcinomas, which is severely decreased due to morphological changes at tumoral level [20]. A similar decrease in vascularity is sometimes encountered in pancreatitis lesions, due to increased fibrosis and the reorganization of the tissue. However, the contrast agent can still achieve similar perfusion levels to surrounding parenchyma, as opposed to the neovascularization specific to pancreatic cancers [10,21]. This visible decrease in contrast uptake is considered by many authors to be the hallmark feature of pancreatic adenocarcinomas, used to differentiate them from pancreatitis masses [3-5,22].

Another advantage of using the referenced approach in describing TICs was represented by the possibility of calculating the time necessary to reach the peak difference in signal, which, for adenocarcinomas, was the time when the parenchyma had the highest value when compared to the tumoral ROI. Our values were somewhat similar to those reported by Kersting *et al*. during CEUS investigations, in which significantly more time passed before peak intensities were reached in pancreatic ductal carcinomas [10].

AUC expresses the differences in amounts of contrast flowing during the investigation in the two specified ROI, as an integral of the sum of median maximum frame intensities, since we calculated the AUC for the reference curves obtained. Hence, we obtained significantly lower AUC for pseudotumoral pancreatitis in which the contrast uptake was similar to that in the healthy pancreas reference ROI, while obtaining a much higher AUC in cases with pancreatic adenocarcinomas.

Previous published studies clearly clarified that pancreatic adenocarcinoma is a hypovascular tumor, although more than 65% of the patients have detectable vessels inside [19,23] Undifferentiated pancreatic tumors might become hypervascular during the natural evolution, thus complicating the differential diagnosis. This situation can be overcome adding another non-invasive technique performed in the same EUS examination which can accurately predict the diagnosis – the real time EUS elastography. To the best of our knowledge, the combination of both methods performed during the same EUS examination has not been tested in large multicentric studies. EUS elastography had already be confirmed as an important non-invasive diagnostic tool in an European multicenter study [24,25]. Recent data on the usefulness of CELMI EUS in comparison with CEHMI EUS found higher sensitivity and specificity for the latter (84.2% and 76.9% versus 89.5% and 92.3%, respectively) [2]. A combination between the two techniques and a third, either elastography or standard B-mode US, did not yield higher diagnostic rates. One similar study combining information provided by contrast enhanced color Doppler EUS and EUS elastography [5] showed specificity and predictive positive values higher than 95%, indicating a high predictability of malignancy in these patients. Although this combination of the methods will not reduce the need for EUS-FNA, it does raise the possibility of referring patients directly for surgery in the presence of resectable focal masses.

## Conclusions

In conclusion, CEH–EUS is a promising method that permits a better differentiation of focal pancreatic masses. As future applications, it can surely be used for patients’ follow-up during chemotherapy or anti-angiogenic treatment. One possible limitation of our study would be the relatively low number of patients included. Even so, the strength of the study in offered by computed analysis of the recorded movies with no human bias introduced and no subsequent knowledge about patients’ history of the doctor selecting the ROI (C.T.S.). Considering the inclusion of the patients belonging of a single tertiary center, large European multicentric studies with adequate power are needed in order to validate the method.

## Competing interests

The authors declare that they have no competing interests.

## Authors’ contributions

DIG and CTS wrote this paper; AS and DIG designed the research and performed the imaging procedures; CTS performed the statistical analysis; TC assisted in the scientific writing of the paper. All authors read and approved the final manuscript.

## Pre-publication history

The pre-publication history for this paper can be accessed here:

http://www.biomedcentral.com/1471-230X/13/2/prepub
